# Case Report: Evidences of myasthenia and cerebellar atrophy in a chinese patient with novel compound heterozygous *MSTO1* variants

**DOI:** 10.3389/fgene.2022.947886

**Published:** 2022-08-11

**Authors:** Liqun Liu, Ruiting Su, Peng Huang, Xingfang Li, Jie Xiong, Yangyang Xiao, Dingan Mao, Lingjuan Liu

**Affiliations:** ^1^ Department of Pediatrics, The Second Xiangya Hospital of Central South University, Changsha, China; ^2^ Department of Pediatric Neurology, Patientren’s Medical Center, Xiangya Hospital of Central South University, Changsha, China; ^3^ Department of Clinical Medicine, Xiangya Medical College, Central South University, Changsha, China

**Keywords:** *MSTO1*, compound heterozygous variants, myasthenia, cerebellar atrophy, mitochondria

## Abstract

Misato Mitochondrial Distribution and Morphology Regulator 1 (MSTO1) is a soluble cytoplasmic protein that regulates mitochondrial dynamics by promoting mitochondrial fusion. Variants in the *MSTO1* gene cause a rare disease characterized by early-onset myopathy and cerebellar ataxia, with almost 30 cases reported worldwide. Here we report a case of a 3-year-old boy with novel heterozygous variants of the *MSTO1* gene (c.1A>G (p.M1?) and c.727G>C(p.Ala243Pro)). Sequencing data and subsequent validation show that the two variants were inherited from the mother and father of the patient (both were heterozygous). The clinical features are infancy-onset mental and motor retardation, language disorder, dysarthria, scoliosis, cerebellar atrophy, tremor, lower-extremity muscle weakness, elevated muscle enzymes, extensive myopathy with chronic atrophy, hyperventilation lungs, and previously unreported hairy back and enlarged gastrocnemius. Finally, novel heterozygous *MSTO1* variants were discovered in this case, which expands the gene spectrum and clinical phenotype of this type of disease, and provides a new direction for future treatment and research. Then we summarize the mutational spectrum, pathological, clinical features and imaging of *MSTO1* variants in a cohort of reported 31 patients and discuss the pathogenesis of *MSTO1* in humans.

## Introduction

Mitochondria, wildly present in most cells (except red blood cells), are involved in the process of adenosine triphosphate (ATP) production, calcium homeostasis, apoptosis and so on (Alston et al., 2021; Ng et al., 2021). Mitochondria are highly dynamic organelles whose morphology, distribution and activity depend on fusion and fission (Chan, 2017). Several neurodegenerative diseases, such as optic atrophy, Charcot-Marie-Tooth disease, Alzheimer’s disease, Parkinson’s disease, and Huntington’s disease, are associated with altered mitochondrial dynamics (Cisneros et al., 2022; Li et al., 2022; Trushina et al., 2022). The prevalence of mitochondrial diseases is approximately 1:2,000, and with recent advances in identification mitochondria DNA, mitochondrial proteins and genetic diagnostics, the number of disease-related variantss in humans is expected to increase substantially (Suomalainen, 2015). The *MSTO1* gene is located on chromosome 1, contains 14 exons, and encodes a protein called protein misato homolog 1. The *MSTO1* gene in humans is highly homologous with that in *drosophila*. Previously, it was found in *drosophila* that nonsense variants in *MSTO1* could lead to irregular chromosome segregation. The application of siRNA to inhibit the expression of MSTO1 *in vivo* could result in mitochondrial fragmentation and aggravate cell death. MSTO1 deficiency leading to human disease was not reported until 2017. MSTO1 is widely distributed in human tissues, and the subcellular localization of the protein studied by using anti-misato antibodies indicates that it is localized to mitochondria, especially the mitochondrial outer membrane. The transfection with Misato siRNA has severe growth defects in HeLa cells. The disappearance of the filamentous mitochondrial network, mitochondrial fragmentation, and ultimately accelerated apoptosis, suggesting that human Misato plays an important role in mitochondrial fusion (Kimura and Okano, 2007). However, the mechanisms of MSTO1 supporting mitochondrial morphogenesis and its potential relevance to human disease remain unclear.

In the present study, we report a patient with early-onset *MSTO1*-related muscular dystrophy who was clinically characterized by infancy-onset mental and motor retardation, scoliosis, tremor, bilateral lower extremity muscle weakness, elevated muscle enzymes, and hairy back. Radiographic evidence of cerebellar atrophy and extensive chronic muscle atrophy, as well as developmental assessment suggesting severe language retardation, further expands the genotype and clinical phenotype of this rare neuromuscular disorder.

## Case presentation

**FIGURE 1:**
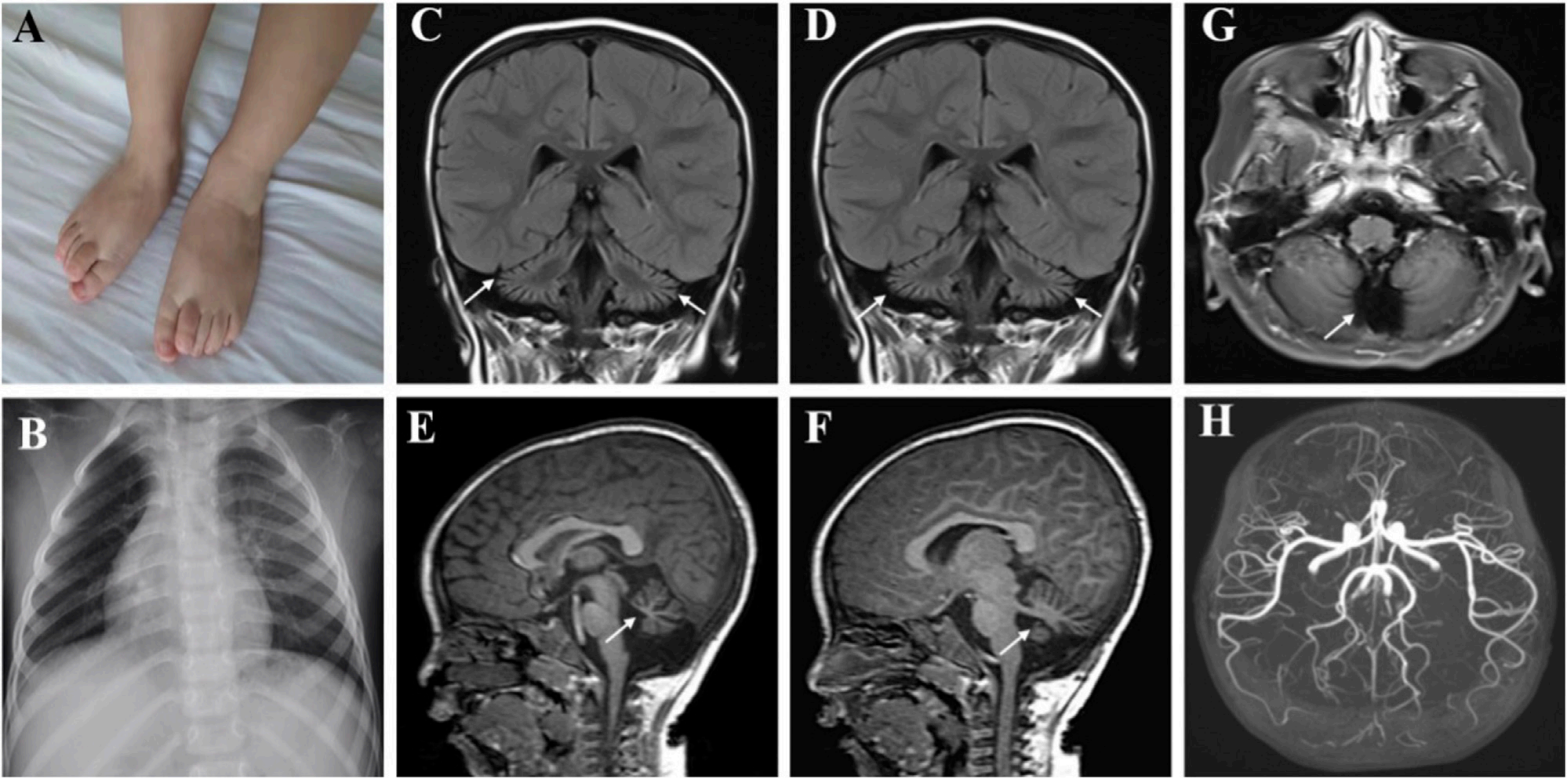
Clinical features of the patient with *MSTO1* variant. The second toes of both feet were upward deformity **(A)**. Chest radiograph showed increased brightness of the right lung, hyperventilation was considered **(B)**. **(C–F)** Brain MRI revealed reduced cerebellar volume, deepened sulci and fissures, and narrowed gyri, the fourth ventricle and occipital cisterna were enlarged **(G)**. MRA was normal **(H)**.

**FIGURE 2:**
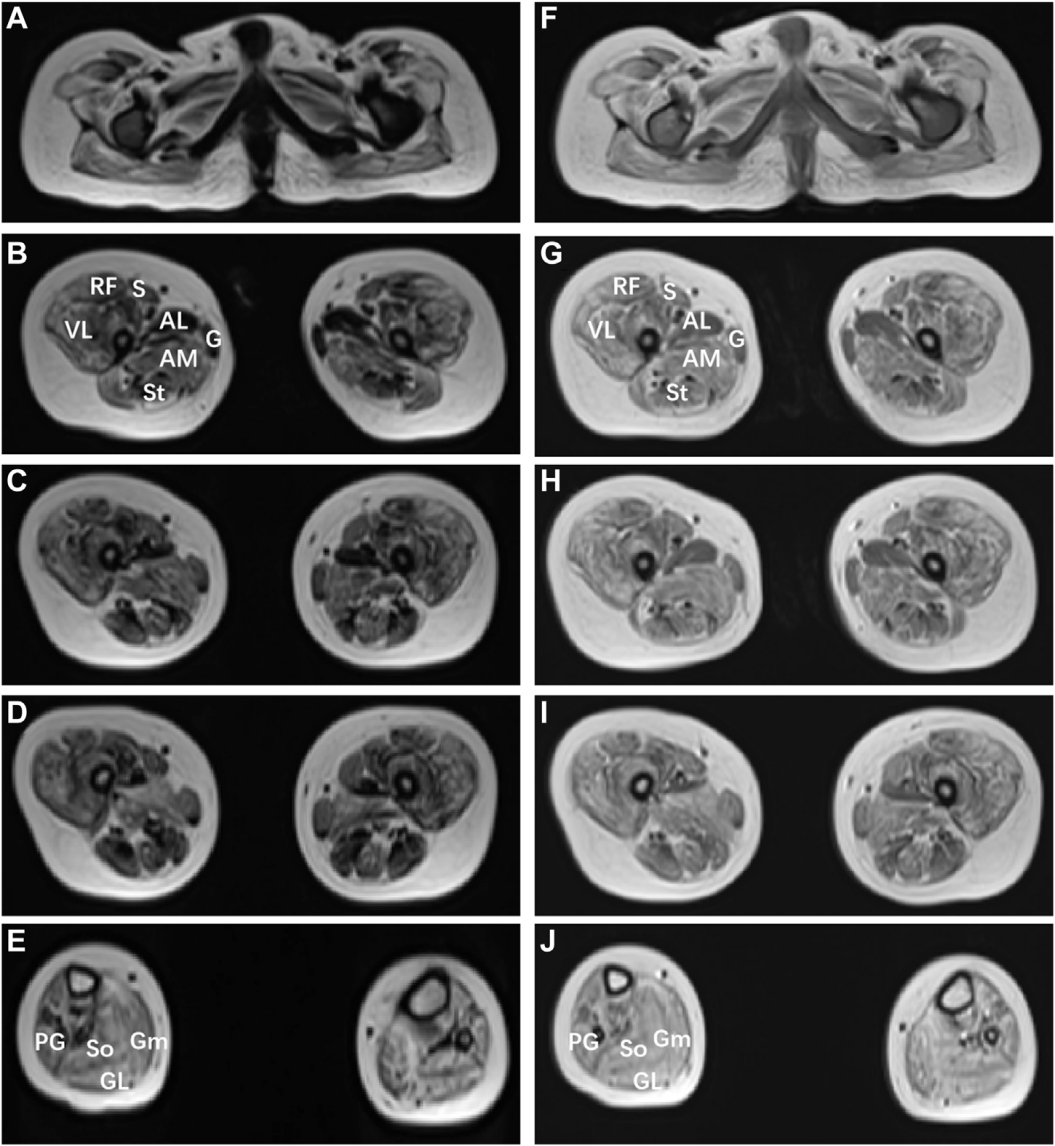
Muscle MRI findings of the patient with *MSTO1* variant. Muscle MRI images of the short T1 signal shadow, uneven T2WI signal in muscle groups of both lower limbs **(A–E)**, and uneven enhancement was observed after enhancement **(F–J)**. The patient shows marked involvement of the glutei within the pelvis **(A,F)**, within the thigh **(B–D)**; **(G–I)**, the pattern of involvement was diffuse, with relative sparing of gracilis **(G)** compared to sartorius (S), and adductor longus (AL) compared to adductor magnus (AM). VL: vastus lateralis; S: soleus; Gm: gastrocnemius medialis, Gl: gastrocnemius lateralis.

The patient could sit at 1.25 years, started to speak and at most two syllables at the age of 1 year and 6 months. The rehabilitation training given to him by the other hospital for half a year had no significant effect. At the age of 3, the patient could sit alone and stand with a handrail, but was not able to stand and walk alone. He could speak two simple syllables. Hand tremors occurred, but lessened when he holding something. He was the first patient of a non-consanguineous family, born without birth trauma and asphyxia, in a mixed feeding way, and supplementary food was added on time. His parents were healthy, denying the familial genetic history and similar medical history. The physical examination showed that the head circumference was 48 cm, he was 87.8 cm tall, and weighed 11.5 Kg, with a sitting height of 51.5 cm. The patient was found to have developmental delay, malnutrition, hairy back, mild scoliosis, and bilateral single transverse palmar creases. Additionally, the muscle strength of both upper extremities was normal, that of both lower extremities was grade 4, the muscle tone was normal, and with enlarged gastrocnemius. There was no edema in the lower extremities. The muscle strength and muscle tone of the limbs were normal, and the second toes of both feet were upward deformity (Figure 1A). Regarding laboratory examinations, plasma muscle enzymes showed creatine kinase (CK) 1164.2 u/l, creatine kinase isoenzyme 47.6 u/l, *α*-hydroxybutyrate dehydrogenase 323.3 u/l, lactate dehydrogenase (LDH) 386.7 u/l, and lactic acid 2.62 mmol/L. There were no obvious abnormalities in the tests for blood routine, liver and kidney function, coagulation function, blood ammonia, immune function and complement, ceruloplasmin, anti-streptolysin (ASO), lupus-associated antibodies, and lymphocyte subsets. Electromyography (EMG) suggested the active myogenic impairment. Chest radiograph showed increased brightness of the right lung, hyperventilation was considered (Figure 1B). The electrocardiogram showed incomplete right bundle branch block. Based on the developmental assessment, adaptability development quotient (DQ) score was 38, suggesting severe developmental delay; gross motor DQ score was 21, indicating very severe developmental delay; fine motor DQ scoring 22, referred to severe developmental delay; language DQ scored 40, namely moderate developmental delay; personal social skills DQ scored 13, meaning moderate developmental delay. He was with ametropia. Brain MRI revealed reduced cerebellar volume, deepened sulci and fissures, narrowed gyri (Figures 1C–F), and enlarged fourth ventricle and occipital cisterna (Figure 1G). There were no abnormalities in the head magnetic resonance angiography (MRA) (Figure 1H) and magnetic resonance venography (MRV) and bilateral hippocampal magnetic resonance spectroscopy (MRS). Moreover, the muscle MRI of both lower extremities showed changes and chronic atrophy of various muscle groups in the lower extremities, and adductor longus (AL) was most seriously affected and muscle atrophy was the most significant (Figures 2A–J). After the doctor detailed the patient’s condition, the family still refused to cooperate with the completion of the muscle biopsy and eye examination. He was given B vitamins to nourish the nerves, oral ATP and coenzyme A, but the symptoms of the patient gradually worsened. Now he was unable to stand up and walk independently. He could only utter two syllables with inaccurate pronunciation.

## Exome analysis

**FIGURE 3:**
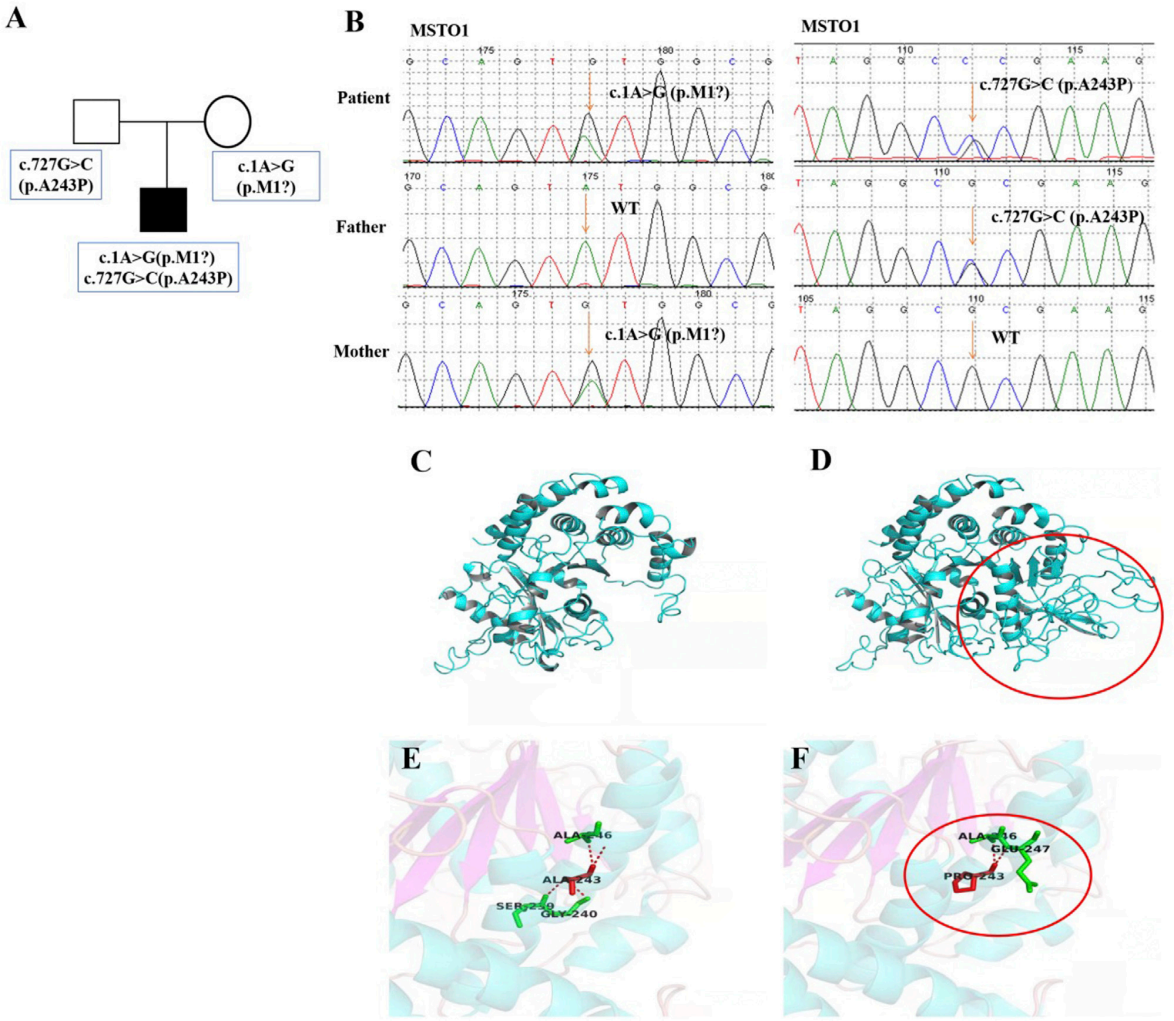
Pedigree features of the patient with *MSTO1* variant. **(A)** Pedigree with the identified a compound heterozygous MSTO1 variants. **(B)** Sequencing verification results showed two heterozygous variants c.1A>G (p.M1?) and c.727G>C (p.A243P) of the *MSTO1* gene were inherited from his parents respectively. c.1A>G variant was located at the first position of the coding sequence, which affected the start codon, and led to abnormal protein structure after variant, especially in the loop structure region. c.727G>C variant could induce a reduction in the number of associated hydrogen bonds between the interior of the protein of the 243rd position and other amino acids from 4 to 2, which was predicted to also affect the protein structure **(D,F)**.

Whole exome sequencing (WES) (completed by Guangzhou Jiajian Medical Laboratory) was performed with the consent of the parents of the proband, and then Sanger sequencing was used to verify the suspected pathogenic loci. The results showed that the proband was detected with compound heterozygous variants of the *MSTO1* gene, c.1A>G (p.M1?) and c.727G>C (p.Ala243Pro). Sequencing data and subsequent validation experiments showed that the two variants were inherited from the mother and father of the patient, respectively (Figures 3A,B), where had not been reported before. After protein structure prediction, it was found that the former was located at the first position of the coding sequence, which might result in absence of protein in case there is no alternative start site. While after the c.727G>C variant occurs, the number of associated hydrogen bonds between the interior of the protein of the 243rd position and other amino acids was reduced from 4 to 2, which was predicted to also affect the protein structure (Figures 3D,F). And, the normal protein structure was showed on Figures 3C,E. The proband’s parents carried heterozygous variants in different loci of the *MSTO1* gene, but both had completely normal phenotypes. Due to the existence of highly-homologous sequences in the *MSTO1* gene, special primers and experimental design were carried out during validation, but it might still fail to distinguish them due to the complexity of the genome. However, combined with the clinical phenotype, imaging features, and genetic testing results of the patient, the diagnosis was clear.

## Discussion

In this paper, we report a patient with infancy-onset compound heterozygous variants in *MSTO1* gene. The patient was clinically featured by early-onset mental and motor retardation, language developmental delay and dysarthria, scoliosis, tremor, muscle weakness, elevated plasma muscle enzymes. EMG suggested myogenic lesions and cerebellar atrophy, overlapping well with previous reports. Besides, the patient also had features such as bilateral single transverse palmar creases, a hairy back, chronic atrophy of muscle groups in both lower extremities, and lung hyperventilation, which have not been reported before, enriching the clinical phenotype of *MSTO1* gene variant. These additional clinical manifestations may be related to mitochondrial dysfunction, considering that MSTO1 has previously been shown to play a critical role in maintaining normal mitochondrial dynamics. And for clinicians, it is very important to understand the full phenotypic spectrum associated with the disease so as to make a correct diagnosis for clinicians. In addition, the genotype of this patient showed two novel heterozygous variants of the *MSTO1* gene, namely, c.1A>G (p. M1?) and c.727G>C (p. Ala243Po), which were inherited from the mother and father, respectively. The variants sites have not been reported yet, and the protein prediction results suggested that the protein phenotype could be affected, further expanding the genotype of MSTO1.

We summarize almost 30 patients reported worldwide so far, as well as sum up their genotypes, clinical features, laboratory examinations, and imaging findings in Supplementary Table S1. The main clinical manifestations were mental and motor retardation, growth restriction, increased CK, skeletal deformity, and retinitis pigmentosa. The *MSTO1* gene was mostly presented with compound heterozygous variant, and only 6 cases (2 families) were homozygous variants. Most of the patients developed the disease in infancy, and 4 of them had symptoms after birth, with the latest onset at age 53. The majority of patients had motor delay as the initial symptom, only 2 patients initially presented with ataxia, and 6 patients had no clinical manifestations temporarily when the genetic diagnosis was clear. 12 patients had growth impairment, and 26 patients showed cognitive deficiency, including learning difficulties and speech delay. Skeletal abnormalities occurred in 9 patients, and the common clinical manifestations were pes planus and mild genu valgus, but about 2/3 of the patients had no skeletal deformities. Pigmentary retinal denaturation was also found in some cases. Plasma CK was elevated in most patients, up to 5200 U/L, while no elevation was observed in a few patients. Imaging mostly suggested cerebellar atrophy, and some cases involved vermis and both hemispheres.

MSTO1 is a soluble cytoplasmic protein whose pathogenic mechanism has not been fully elucidated. Existing studies suggest that MSTO1 interacts with the mitochondrial outer membrane during mitochondrial fusion, and then participates in the formation of mitochondrial networks (Schultz-Rogers et al., 2019). Misato1 plays a critical role in regulating mitochondrial morphology and distribution as well as mitochondrial dynamics. The pathogenic variant of the *MSTO1* gene could cause abnormal expression of Misato protein, lysis, fragmentation and disappearance of reticular structure of cell mitochondria, thereby reducing mitochondrial DNA and eventually causing mtDNA depletion syndrome (Donkervoort et al., 2019). On average, each cell has 100–1000 copies of mtDNA, which can encode 13 proteins necessary for oxidative phosphorylation, thus affecting the mitochondrial respiratory chain and the production of ATP. Cells must maintain a certain level of mtDNA to ensure the consumption of their energy metabolism. If the total amount of mtDNA is reduced, multi-system abnormalities will appear clinically, and organs with greater energy requirements (such as brain tissue and muscle) are more likely to be involved. Therefore, most cases reported so far have the following clinical features: mental and motor retardation, mental illness, cerebellar ataxia, muscle involvement (increased muscle enzymes, dystonia, muscle weakness and atrophy) and liver disease (hepatomegaly, liver failure and severe lactic acidosis) (Cheema et al., 2021).

MSTO1 is currently considered to be autosomal recessive, and its pathogenic missense variants often occur in tubulin domains, suggesting that these regions are critical for normal protein function and stability. Nasca et al. (Nasca et al., 2017) hypothesized that the decreased protein expression in these patients was due to instability caused by missense variants. While biallelic loss-of-function variants could be lethal, missense variants that retained some function could cause disease states. This idea was supported by research in *Drosophila*. In contrast, in the cases reported by Gal et al. (Gal et al., 2017) most patients had no symptoms of cerebellar atrophy and retinitis pigmentosa, which did not overlap well with the clinical symptoms of other cases, so it was proposed whether MSOT1 has an autosomal dominant inheritance pattern, and the clinical phenotype may depend on the location of the variant or other underlying genetic factors. In this paper, we report a case of neuromuscular disease caused by a compound heterozygous variant of the *MSTO1* gene. WES analysis indicates that there were 2 novel variants, c.1A>G (p.M1?) and c.727G>C (p.Ala243Pro), respectively. The clinical symptoms of the patient overlapped with those of most patients, but an increasingly hairy back and enlarged gastrocnemius have not been reported before. These two variants of the *MSTO1* gene are unreported in relevant clinical cases, and were considered pathogenic variants after protein structure prediction. Consistent with a previous report (Ardicli et al., 2019), the patient’s muscle MRI shows extensive involvement and chronic atrophy of both lower limb muscles, and gracilis and AL were the most severely affected muscles. Compared with a muscle biopsy, MRI is less invasive and more easily accepted by patients and their families, and has good guiding significance for the location and qualitative of muscle lesions. It is recommended to complete MRIs as many as possible for patients with myopathy. Additionally, most reported cases had symptoms of retinitis pigmentosa, but regrettably, the current patient’s parents refuse to perform the eye examination and muscle biopsy, and the occurrence of eye complications could not be ruled out, only ametropia was detected in the child. It is needed to keep regular track of the patient’s condition, and persuade him and his family into completing the relevant examinations.

## Conclusion

In conclusion, this report further expands the phenotype and genetics of the disease, and this case adds to our current knowledge of the association of mitochondrial myopathy with pathogenic variants in the *MSTO1* gene, puts forward previously undescribed novel phenotypic features associated with *MSTO1*-related disorders, and also provides new ideas for future treatments and research directions for this disease. In the future, we expect to collect more relevant cases to further elucidate and study the other roles of MSTO1 in mitochondrial dynamics and related mitochondrial diseases.

## Data Availability

The original contributions presented in the study are included in the article/Supplementary Material, further inquiries can be directed to the corresponding author.

## Abbreviations

ASO, anti streptolysin; ATP, adenosine triphosphate; CK, creatine kinase; DQ, development quotient; EMG, Electromyography; Gl, gastrocnemius lateralis; Gm, gastrocnemius medialis; LDH, lactate dehydrogenase; MRA, magnetic resonance angiography; MSTO1, misato homolog 1; MRS, magnetic resonance spectroscopy; MRV, magnetic resonance venography; So: soleus; VL, vastus lateralis; WES, Whole exome sequencing.
