# Supplementary figures and images for: Case Report: Evidences of myasthenia and cerebellar atrophy in a chinese patient with novel compound heterozygous MSTO1 variants
Source: Front Genet. 2022 Aug 11;13:947886. doi: 10.3389/fgene.2022.947886 (PMC9402982; doi:10.3389/fgene.2022.947886)

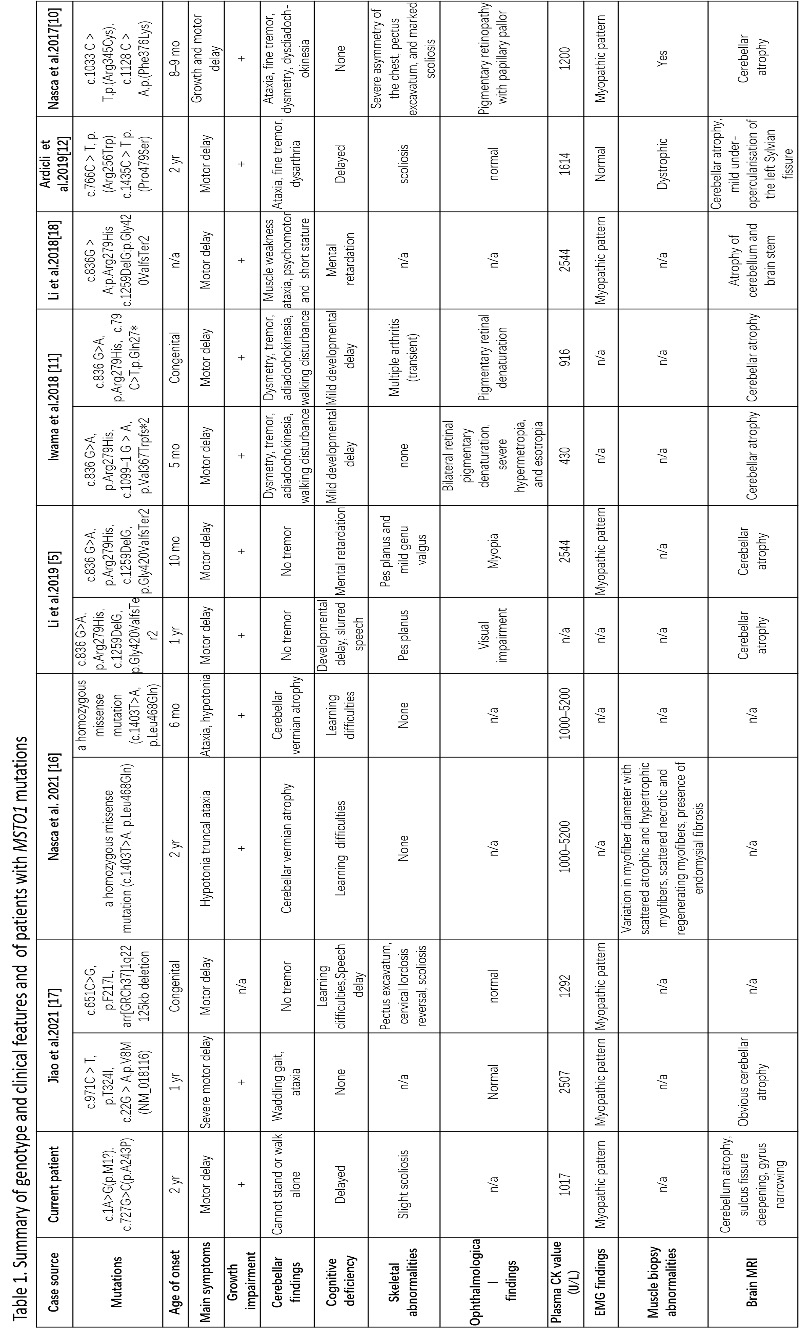


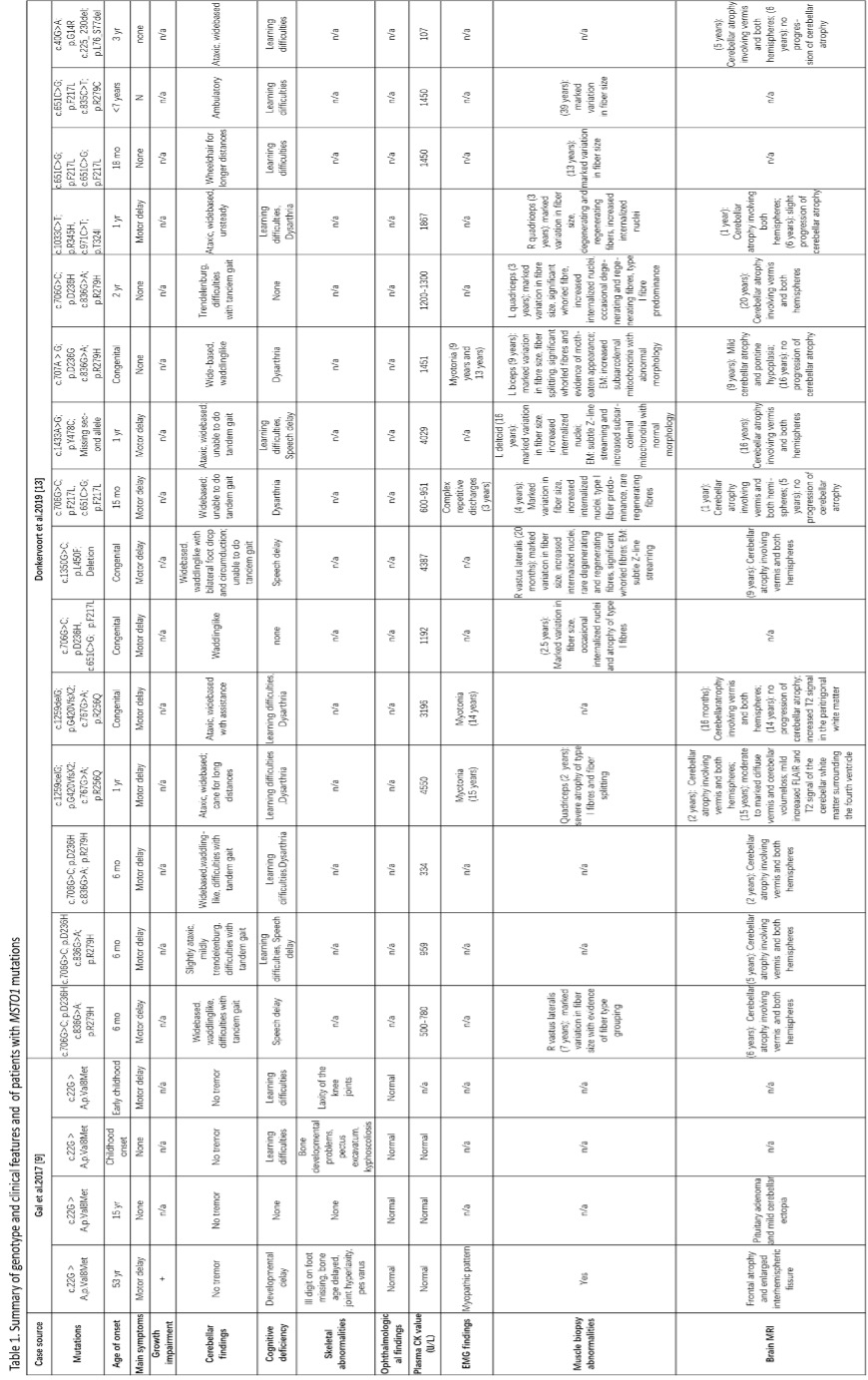

Supplement: Supplementary file 1 [file Table1.docx]
